# Identification of Potential Roles of Bestrophin 3 in the Growth Performance of Ortiental River Prawn *Macrobrachium nipponense* by RNA Interference

**DOI:** 10.3390/ijms27125338

**Published:** 2026-06-13

**Authors:** Shubo Jin, Zijian Gao, Hongtuo Fu, Yiwei Xiong, Hui Qiao, Wenyi Zhang, Sufei Jiang

**Affiliations:** 1 Key Laboratory of Freshwater Fisheries and Germplasm Resources Utilization, Ministry of Agriculture and Rural Affairs, Freshwater Fisheries Research Center, Chinese Academy of Fishery Sciences, Wuxi 214081, China; jinsb@ffrc.cn (S.J.); fuht@ffrc.cn (H.F.); xiongyw@ffrc.cn (Y.X.); qiaoh@ffrc.cn (H.Q.); 2 Wuxi Fisheries College, Nanjing Agricultural University, Wuxi 214081, China; gaozijiangenomics@163.com

**Keywords:** crustacean, qPCR, body mass gain, growth-related SNPs, marker-assisted selection

## Abstract

*Macrobrachium nipponense* is an economically important freshwater prawn species in China, where larger individuals have higher commercial value than smaller ones. Previous studies indicated that bestrophin 3 (*BEST3*) may play a regulatory role in the growth performance of this species. Therefore, the present study investigated the potential functions of the *BEST3* gene in the growth of *M. nipponense* by using quantitative real-time PCR (qPCR) and RNA interference (RNAi), and also searched for growth-related single-nucleotide polymorphisms (SNPs) within this gene. qPCR results revealed that *Mn-BEST3* expression was widely detected across all tested tissues, suggesting that this gene may serve multiple functions in *M. nipponense*. Notably, its highest expression was observed in muscle tissue, which was significantly greater than that in all other tested tissues (*p* < 0.05), implicating a potential role for this gene in growth regulation. Further qPCR analysis confirmed that the synthesized *dsBEST3* effectively reduced *Mn-BEST3* expression. The body mass gain percentage in the *dsBEST3*-injected group was significantly lower than that in the *dsGFP*-injected control group, with differences becoming significant from Day 12 onward in both males and females (*p* < 0.05). These findings indicate that *Mn-BEST3* plays a positive role in regulating growth in *M. nipponense*. Finally, three SNPs were identified in the coding region of this gene. The associations of these three SNPs with growth performance, including body weight and total length, were further validated using 50 male and 50 female prawns derived from a full-sib family at approximately 5 months post-hatching. Among them, one SNP (S31_23192836) was found to be associated with growth performance in both male prawns and female prawns. Overall, this study confirmed the positive regulatory role of *BEST3* in the growth of *M. nipponense* and identified growth-related SNPs within this gene. These results improve our understanding of the molecular mechanisms underlying growth regulation and support the production of populations with superior growth traits through marker-assisted selection.

## 1. Introduction

The oriental river prawn, *Macrobrachium nipponense*, is a commercially important freshwater species widely found across China, Japan, Korea, Vietnam, Myanmar, and Iran [1,2,3,4,5]. It mainly lives in freshwater environments such as rivers, lakes, reservoirs, and ponds, but also tolerates low-salinity conditions well, so it is commonly seen in estuarine areas as well. Because of its fast growth, high reproductive capacity, and good adaptability to different farming conditions, *M. nipponense* has become one of the most economically valuable freshwater prawn species in China. In 2024, the annual production of this species reached about 230,000 metric tons, ranking fourth among freshwater commercial prawn species in China. This highlights its important role in the economic performance of the aquaculture industry [6].

Growth performance is a key commercial trait affecting the economic value of aquatic animals, given that larger individuals are typically worth more than smaller ones [7]. For this reason, producing populations with improved growth traits through genetic selection has long been a major goal in aquaculture. In *M. nipponense*, growth performance is known to be influenced by multiple factors, including stocking density [8,9,10], nutrition [11,12,13], molting [14], and environmental conditions [15,16]. As an important economical trait, growth is also influenced by genetic factors. To identify growth-related genes in this species, a genome-wide association study combined with transcriptome analysis was carried out [17]. In addition, transcriptome analysis revealed that an actin-like gene was upregulated in fast-growing individuals, suggesting that this gene may be involved in the regulation of growth in *M. nipponense* [16]. Subsequent qPCR and RNAi analysis demonstrated that this gene positively regulates growth in *M. nipponense*, confirming the accuracy of Reference 16 in screening growth-related genes in this species [18].

Previous transcriptome profiling analysis showed that bestrophin 3 (*BEST3*) is markedly up-regulated in fast-growing individuals of *M. nipponense*, suggesting a regulatory role for this gene in growth regulation [17]. Bestrophins (*Best*) are a family of transmembrane proteins that act as Ca^2+^-activated Cl^−^ channels in the plasma membranes of epithelial and non-epithelial cells [19]. The *BEST3* gene encodes bestrophin 3, one of the bestrophin anion channels. This protein is a transmembrane protein containing a region of homology that is rich in aromatic amino acid residues and includes an invariant R-F-P (arginine-phenylalanine-proline) motif [20]. In mammals, *BEST3* can function as a Ca^2+^-activated Cl^−^ channel in cardiac [21] and smooth muscle tissues [22]. Ca^2+^-activated Cl^−^ channels (CaCC) are abundantly present in arterial smooth muscle cells (ASMCs). They are key ion channels that function in maintaining the membrane polarization state of ASMCs and regulating the contractility of both these cells and arteries [23]. In addition, *BEST3* has been proposed as a candidate gene for mandibular prognathism, a condition marked by excessive endochondral growth of the mandibular condyle [24].

Over the past few decades, a number of certified improved varieties of aquatic species have been developed through conventional breeding programs, with growth performance serving as the main selection trait. These varieties have been officially certified by the Ministry of Agriculture of China, and their promotion and use have greatly contributed to the sustainable development of aquaculture [25]. Traditional breeding strategies include mass selection, family selection, and hybridization. These approaches rely on phenotypic and pedigree information at the individual or family level, but they are often constrained by high labor costs and long generation intervals [26,27]. By contrast, marker-assisted selection (MAS) offers advantages over conventional methods. It allows for more efficient identification of candidate accessions for phenotypic evaluation, while also reducing the resources needed for seedling maintenance, including both cost and space [28]. Thus, there is an urgent need to identify molecular markers associated with growth in *M. nipponense* to support selective breeding of strains with better growth performance.

In this study, we used quantitative real-time PCR (qPCR) and RNA interference (RNAi) to explore whether *BEST3* plays a role in regulating growth performance in *M. nipponense*. We also screened for growth-related single-nucleotide polymorphisms (SNPs) within this gene by PCR amplification and Sanger sequencing. Our findings help clarify the molecular mechanisms underlying the growth in *M. nipponense* and may support the use of MAS to breed populations with better growth traits in this species.

## 2. Results

### 2.1. Sequence Analysis

**Figure 1:**
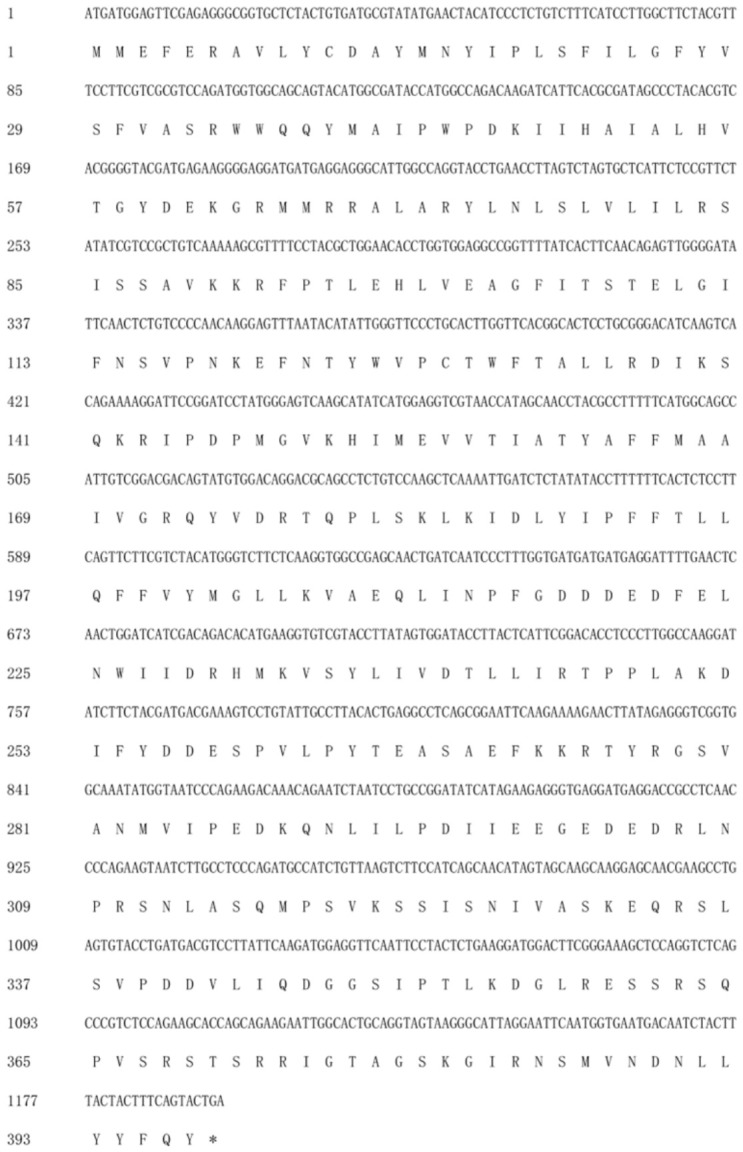
The open reading frame (ORF) sequence of the *Mn-BEST3* gene is shown, with both the nucleotide sequence and the deduced amino acid sequence presented in the 5′→3′ direction. In the deduced amino acid sequence, each amino acid is represented by its single-letter uppercase code. The start codon (ATG) and the stop codon (TAA, marked by an asterisk) are clearly indicated.

**Figure 2:**
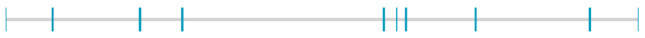
The genome structure of *Mn-BEST3*. The blue boxes indicated the exons.

**Figure 3:**
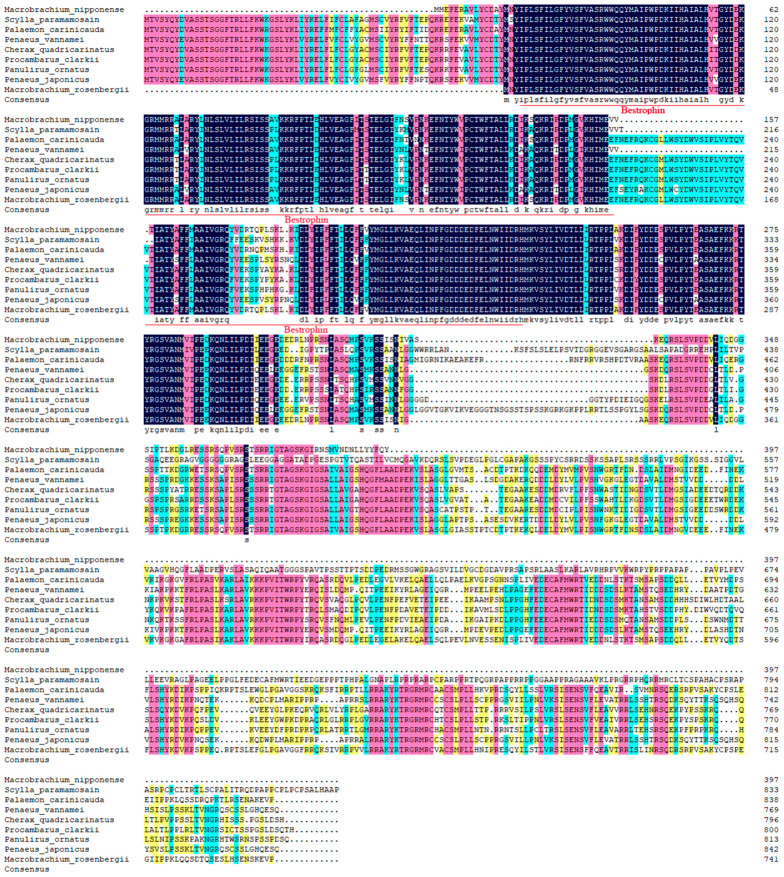
Sequence alignment and structural information of the *BEST3* protein from *M. nipponense*. Red line indicated the conserved bestrophin domain within the *BEST3* protein. Black, pink, blue, and yellow indicated 100%, ≥75%, ≥50%, and ≥33% identity between species, respectively.

**Figure 4:**
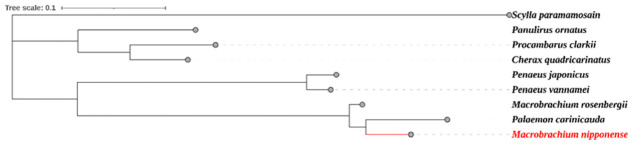
Phylogenetic tree analysis of BEST3 protein in crustaceans. *Macrobrachium rosenbergii* (XP_066944196.1); *Scylla paramamosain* (KAK8379233.1); *Palaemon carinicauda* (XP_068205836.1); *Penaeus vannamei* (XP_069969864.1); *Cherax quadricarinatus* (XP_069940637.1); *Procambarus clarkii* (XP_045599924.1); *Panulirus ornatus* (XP_071545299.1); *Penaeus japonicus* (XP_042887574.1).

The coding region of *Mn-BEST3* was 1194 bp in length, encoding a protein of 397 amino acids (Figure 1). The predicted molecular weight and isoelectric point of the encoded protein were 45.61 kDa and 6.561, respectively. The genomic sequence of this gene spaned 43,543 bp and was located on Chromosome 31, between positions 23,166,703 bp and 23,210,245 bp. The gene consisted of 10 exons and 9 introns (Figure 2; Table 1). Within the deduced protein, a conserved bestrophin functional domain was predicted, spanning amino acid residues from 15 to 231 (Figure 3). Furthermore, Blastx analysis against the NCBI database indicated that the *Mn-BEST3* amino acid sequence shareed high identity with orthologs from other shrimp species. It exhibits the highest identity with *Macrobrachium rosenbergii* (90.75%), followed by *Scylla paramamosain* (83.69%), *Palaemon carinicauda* (82.77%) and *Penaeus vannamei* (80.31%). Maximum likelihood phylogenetic analysis revealed that *Mn-BEST3* is most closely related to the ortholog from *P. carinicauda*, forming a clade with *M. rosenbergii* (Figure 4).

### 2.2. qPCR Analysis

**Figure 5:**
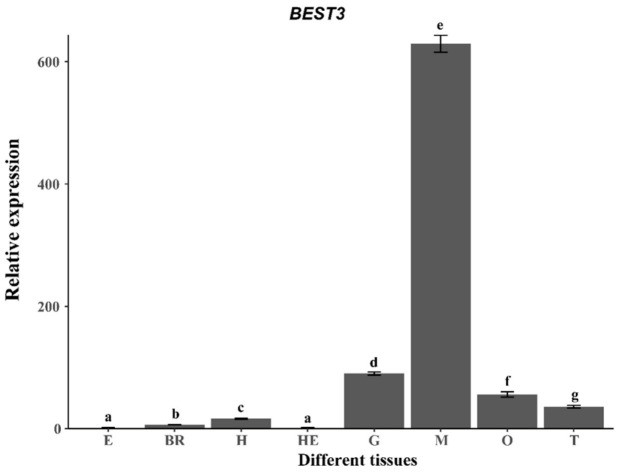
qPCR analysis was used to measure the relative expression levels of the *BEST3* gene across various mature tissues of *M. nipponense*. The EIF gene served as an internal control for data normalization. All data are shown as mean ± standard deviation (SD, n = 6). Different lowercase letters indicated statistically significant differences in *BEST3* expression among tissues (*p* < 0.05). E, eyestalk; BR, brain; H, heart; HE, hepatopancreas; G, gill; M, muscle; O, ovary; T, testis.

qPCR analysis was performed to examine the mRNA expression levels of *Mn-BEST3* in various tissues (Figure 5). Among the tested tissues, muscle exhibited the highest expression of *Mn-BEST3*, which was significantly higher than that in all other tissues (*p* < 0.05), whereas the hepatopancreas showed the lowest expression. Moderate expression levels were observed in the gill, ovary, and testis. Specifically, the mRNA expression levels in muscle, gill, ovary, and testis were 617.2-fold, 64.3-fold, 45.3-fold, and 33.6-fold higher, respectively, than that in the hepatopancreas.

### 2.3. RNAi Analysis

**Figure 6:**
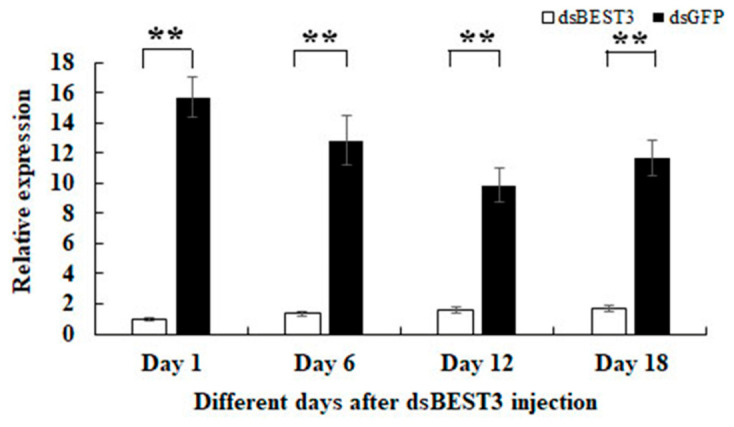
The time-course interference efficiency of *dsBEST3* was assessed using qPCR. Gene expression levels were normalized to the reference gene *EIF* and presented as mean ± SD (n = 3). Double asterisks (**) indicate a highly significant difference (*p* < 0.01) between the *dsBEST3*-treated group and the *dsGFP* control group at matching time points.

RNAi was employed to investigate the potential role of *Mn-BEST3* in the growth performance of *M. nipponense*. The mRNA expression levels of *Mn-BEST3* following injection of *dsBEST3* or *dsGFP* (control) were assessed using qPCR. Compared to the *dsGFP*-injected group on the corresponding days, qPCR analysis revealed that *Mn-BEST3* mRNA expression in the *dsBEST3*-injected prawns was significantly reduced to 93.64%, 89.35%, 84.40%, and 85.57% at days 1, 6, 12, and 18 post-injection, respectively (*p* < 0.05) (Figure 6).

**Figure 7:**
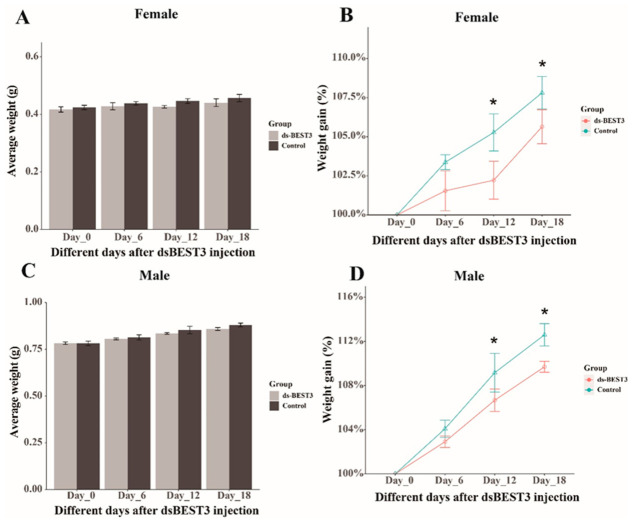
Effect of *dsBEST3* injection on weight gain in *M. nipponense*. Body weight changes were recorded over time after *dsBEST3* or *dsGFP* (control) injection. At identical time points, significant differences between groups are denoted by * (*p* < 0.05). (**A**) Female weight gain. (**B**) Female percent body mass increase. (**C**) Male weight gain. (**D**) Male percent body mass increase.

On Day 0, the initial body weights of female *M. nipponense* in the *dsBEST3*-injected group and the *dsGFP*-injected control group were 0.417 ± 0.03 g and 0.424 ± 0.05 g, respectively. In the *dsGFP*-injected control group, the body weights increased to 0.438 ± 0.06 g, 0.446 ± 0.06 g, and 0.457 ± 0.06 g at Day 6, Day 12, and Day 18, respectively. At the same time points, the body weights in the *dsBEST3*-injected group were 0.423 ± 0.04 g, 0.426 ± 0.04 g, and 0.440 ± 0.05 g, respectively (Figure 7A). When expressed as the percentage increase in mass relative to Day 0, female prawns in the *dsGFP*-injected control group exhibited gains of 3.30%, 5.19%, and 7.78% on Days 6, 12, and 18, respectively. In contrast, the *dsBEST3*-injected group showed mass increases of 1.44%, 2.16%, and 5.51% at the corresponding time points. The percentage mass increase differed significantly between the *dsGFP*-injected control group and the *dsBEST3*-injected group starting from 12 days post-injection (*p* < 0.05) (Figure 7B).

A similar trend in weight gain was observed in male prawns. The initial body weights of male *M. nipponense* in the *dsBEST3*-injected group and the *dsGFP*-injected control group were 0.780 ± 0.10 g and 0.781 ± 0.11 g, respectively. Subsequently, at Day 6, Day 12, and Day 18, the body weights in the *dsGFP*-injected control reached 0.814 ± 0.12 g, 0.855 ± 0.11 g, and 0.881 ± 0.13 g, respectively, while those in the *dsBEST3*-injected group were 0.805 ± 0.11 g, 0.834 ± 0.13 g, and 0.858 ± 0.13 g at the corresponding time points (Figure 7C). The percentage increases in mass relative to Day 0 in the *dsGFP*-injected control group were 4.23%, 9.48%, and 12.80% at Days 6, 12, and 18, respectively. Over the same period, the *dsBEST3*-injected group exhibited corresponding mass increases of 3.21%, 6.92%, and 10.00%. A statistically significant difference in the percentage mass increase between the *dsGFP*-injected control group and the *dsBEST3*-injected group was observed from 12 days post-injection onward (*p* < 0.05) (Figure 7D).

In female prawns, the daily weight gain at Day 18 post-injection was 3.179 ± 0.919‰ in the *dsBEST3*-injected group and 4.293 ± 1.831‰ in the *dsGFP*-injected control group, and this difference was statistically significant (*p* < 0.05). Similarly, in male prawns, the daily weight gain at Day 18 post-injection was 5.384 ± 0.274‰ in the *dsBEST3*-injected group and 6.993 ± 0.563‰ in the *dsGFP*-injected control group, which also showed a significant difference (*p* < 0.05) (Table 2).

### 2.4. Identification of Growth-Related SNPs Within BEST3

A total of 3 SNPs were identified within the coding regions of *Mn-BEST3*. Their observed heterozygosity ranged from 0.311 to 0.396, expected heterozygosity ranged from 0.292 to 0.393, and the polymorphism information content ranged from 0.247 to 0.316. All of these loci were synonymous mutation sites (Table 3).

Among these three SNPs, S31_23192836 (located at the 196th amino acid of this gene) was identified to regulate growth in both male and female prawns (Table 4). A total of 46 female individuals were successfully sequenced, including 22 with the TT genotype, 14 with the TG genotype, and 10 with the GG genotype. The average body weight and full length of the TT genotype were 1.117 g ± 0.504 and 46.237 mm ± 6.100, respectively, which were significantly greater than those of the TG genotype (0.897 g ± 0.429 for body weight and 43.692 mm ± 6.350 for full length) and the GG genotype (0.500g ± 0.190 for body weight and 38.057 mm ± 5.155 for full length) (*p* < 0.05). In male prawns, sequencing was successfully performed on 45 individuals, among which 25 carried the TT genotype, 13 carried the TG genotype, and 7 carried the GG genotype. The TT genotype exhibited significantly higher average body weight (2.500 g ± 0.682) and full length (59.532 mm ± 5.635), compared to the TG genotype (1.699 g ± 0.910 for body weight; 52.919 mm ± 8.475 for full length) and the GG genotype (1.057 g ± 0.142 for body weight; 47.097 mm ± 1.539 for full length), with all differences being statistically significant (*p* < 0.05).

## 3. Discussion

In mammals, *BEST3* functions as a Ca^2+^-activated Cl^−^ channel and plays an essential role in maintaining the membrane polarization state of ASMCs, as well as in regulating the contractility of both ASMCs and arteries [23]. Growth is the primary commercial trait targeted during genetic improvement programs in aquatic animals. Previous transcriptome profiling has suggested that *BEST3* may also be involved in regulating the growth of *M. nipponense* [17]. Therefore, the present study aimed to investigate the potential functions of *BEST3* in the growth regulation of this species, and to identify growth-related SNPs within this gene, thereby facilitating the production of populations with superior growth traits through marker-assisted selection.

The expression of *BEST3* has been characterized in several mammalian species. In human tissues, *BEST3* is strongly expressed in skeletal muscle, with weaker expression detected in bone marrow, testis, and retina [20]. Its expression has also been observed in human cartilage [29]. Immunofluorescence staining revealed *BEST3* expression in the plasma membrane, nuclei, and intracellular compartments of rat kidney cortex sections [22]. Furthermore, a muscle transcriptome profiling analysis showed that *BEST3* expression levels are higher in the larger-bodied cattle species (*Bos frontalis*) than in the common domestic cattle (*Bos taurus*), suggesting that this gene may be involved in growth regulation in cattle [30]. However, to the best of our knowledge, the expression of this gene has not been investigated in any aquatic animal species. The present study revealed that *BEST3* exhibits the highest expression level in muscle tissue. Genes expressed in muscle tissue influence the growth performance of aquatic animals through various molecular and physiological mechanisms. For example, Insulin-like growth factor-1 (IGF-1) and IGF-2, along with their receptors and binding proteins, are expressed in muscle and promote protein synthesis while inhibiting protein degradation, thereby facilitating muscle hypertrophy and hyperplasia [31,32]. Additionally, the myogenic regulatory factor family genes directly control the proliferation, differentiation, and fusion of muscle stem cells into myofibers [33,34]. The expression levels of these genes are closely associated with myofiber number and diameter, ultimately determining individual body size and growth rate. A previous study identified that an actin-like gene was highly expressed in muscle tissue and exerted positive regulatory effects on the growth of *M. nipponense* [18]. Similarly, the high expression of the *BEST3* gene in muscle tissue suggests that this gene may also be involved in regulating growth traits in *M. nipponense*.

RNAi functions as a conserved cellular defense and regulatory mechanism. It utilizes small RNA molecules as guides to specifically recognize and degrade complementary messenger RNA (mRNA) transcripts, thereby silencing gene expression at the post-transcriptional level. RNAi has since been identified across diverse eukaryotic lineages, including crustaceans, where it plays critical roles in antiviral immunity, transposable element suppression, developmental regulation, and physiological adaptation [35,36,37]. Knockdown of *BEST3* led to a reduction in cell number as a consequence of cell death in rat kidney proximal tubule cells under conditions of endoplasmic reticulum stress [22]. However, to the best of our knowledge, the potential functions of this gene have not yet been analyzed in any aquatic animal species using RNAi. The RNAi technique has been well established in *M. nipponense* [38], and the functional roles of numerous genes have been successfully investigated through RNAi analysis in this species [39,40,41,42,43,44]. In the present study, the synthesized *dsBEST3* efficiently knocked down *Mn-BEST3* expression, with a significant reduction of 81–95% relative to the *dsGFP*-injected controls (*p* < 0.05). In both sexes, injection of *dsBEST3* led to impaired growth performance in *M. nipponense*, with a notable decrease in body weight gain observed from Day 12 post-injection. Taken together, these findings demonstrate that this gene positively regulates growth performance in this species. Interestingly, *Mn-BEST3* expression in the *dsGFP* group did not show a progressive increase with increasing individual body weight. Transcriptomic analysis revealed that this gene is highly expressed in larger individuals, indicating its high expression in fast-growing *M. nipponense*. However, this observation does not imply that *Mn-BEST3* expression gradually rises as body weight increases. Additionally, *Mn-BEST3* expression is likely regulated by both endogenous biological rhythms and exogenous environmental cues [18].

SNPs are widely distributed across the genomes of diverse organisms. Mutations occurring in coding regions are known to alter protein structure and function [45,46]. Associations between SNPs and key traits have been documented in *M. nipponense*, regarding hypoxia resistance [47], sexual maturation [48], and growth-related traits [18]. In the present study, three SNP loci were identified within the coding region of the *BEST3* gene in *M. nipponense*, among which S31_23192836 was identified to be associated with growth performance. In both male and female prawns, individuals carrying the T allele demonstrated significantly greater average body weight and total length relative to those with other genotypes. These findings suggest that the T allele significantly promotes growth performance in this species.

## 4. Materials and Methods

### 4.1. Tissue Collection

All prawns were collected from the Dapu Breeding Base in Wuxi, China (120°13′44″ E, 31°28′22″ N), consisting of 286 healthy individuals for functional analysis of *Mn-BEST3* and 100 full-sibs (50 males and 50 females) for SNP selection within the *Mn-BEST3* (Table 5). All prawns were reared in a pond under conditions suitable for the growth of *M. nipponense*, with water temperature ranging from 25 °C to 30 °C and dissolved oxygen concentration kept above 6.0 mg/L [25]. During the rearing period, feeding was conducted twice daily. The prawns used for sequence verification, qPCR analysis, and SNP screening were collected at approximately 5 months post-hatching, as opposed to those used for RNAi analysis, which were collected at approximately 2 months post-hatching. All prawns were acclimatized for three days under controlled laboratory conditions prior to the experiments. During this period, the water temperature was kept at 26.0 ± 1.2 °C, and the dissolved oxygen concentration remained above 6.0 mg/L.

To verify the ORF sequence of *Mn-BEST3*, muscle tissue was dissected from ten randomly selected individuals. A total of 36 *M. nipponense* individuals, consisting of 18 males and 18 females, were collected for qPCR analysis. The mature tissues examined included eyestalk, brain, heart, hepatopancreas, gill, muscle, testis, and ovary. One biological replicate for testis and ovary was generated by pooling samples from three individuals of the respective sex. For the remaining tissues, samples were taken separately from three male and three female individuals, and then pooled to form one biological replicate. Six such replicates were prepared for each tissue. For the RNAi experiment, a total of 240 prawns (120 males and 120 females) were used. Following injection with either double-stranded GFP (*dsGFP*) or double-stranded *BEST3* (*dsBEST3*), muscle tissue was dissected and collected for qPCR analysis. Additionally, full-sibling individuals were subjected to body weight and length measurements, and their muscle tissue was collected for SNP identification. To prevent RNA degradation, all collected samples were immediately flash-frozen in liquid nitrogen and subsequently stored at −80 °C.

### 4.2. Annotation and Comparison of Mn-BEST3

The full-length cDNA sequence of *Mn-BEST3* was obtained by integrating data from the *M. nipponense* genome database (accession number: GCA_015104395.2) and muscle transcriptome datasets (accession numbers: SRX25177010–SRX25177021).

To confirm the sequence, RNAiso Plus reagent (TaKaRa, Dalian, China) was used to extract the total RNA from each muscle sample. RNA concentrations were determined with a spectrophotometer (Eppendorf, Hamburg, Germany), while RNA integrity was assessed via agarose gel electrophoresis. First-strand cDNA was synthesized from approximately 1 µg of total RNA per sample by reverse transcription with the iScript™ cDNA Synthesis Kit (Bio-Rad, Hercules, CA, USA). To ensure sequence accuracy, the obtained sequence was experimentally validated using three specific primer pairs (Table 6) by PCR amplification, with the synthesized cDNA serving as templates. PCR was carried out in a 25 μL reaction mixture containing 0.5 μL each of forward and reverse primers (10 μM), 1 μL cDNA template, 19.9 μL DEPC-treated water, 2.5 μL 10× PCR supermix, 0.5 μL dNTP mix (10 mM), and 0.1 μL Taq DNA polymerase. The thermal cycling program was as follows: initial denaturation at 95 °C for 3 min; 35 cycles of 95 °C for 30 s, 62 °C for 60 s, and 72 °C for 30 s; followed by a final extension at 72 °C for 10 min. The resulting PCR products were then sequenced by Shanghai Shenggong Bioengineering Technology Service Co., Ltd. (Shanghai, China) on an ABI 3730 automated DNA sequencer (Invitrogen Biotechnology Co., Ltd., Carlsbad, CA, USA).

The ORF of *Mn-BEST3* was identified using the online tool ORF-FINDER [49]. The corresponding cDNA sequence was then translated into an amino acid sequence, and the result was visualized with DNAman software (version 6.0) [50]. Multiple sequence alignment of *BEST3* protein sequences from different species was performed using ClustalW (version 2.0) [51]. Based on the aligned sequences, a phylogenetic tree was subsequently reconstructed with MEGA software (version 11) [52] using the maximum likelihood method. Nodal support was assessed by 1000 bootstrap replicates, and the resulting bootstrap values are displayed at the respective nodes.

### 4.3. qPCR Analysis

In the present study, the mRNA expression levels of *Mn-BEST3* were quantified in various mature tissues using qPCR. The procedures for RNA extraction and cDNA synthesis were as described in detail in Section 4.2.

QPCR was carried out on a Bio-Rad iCycler iQ5 system using SYBR Green fluorescence detection. The experimental procedure followed the protocols described in previous studies [53]. QPCR analyses were carried out in a 25 μL reaction mixture consisted of 12.5 μL of 2× Ultra SYBR Mix (CWBIO), 0.5 μL each of the forward and reverse primers (10 μM; see Table 4), 1 μL of cDNA template, and 10.5 μL of nuclease-free water. The thermal cycling conditions were set as follows: initial denaturation at 95 °C for 10 min, then 40 cycles of 95 °C for 15 s and 60 °C for 1 min. All qPCR assays were conducted in triplicate for each tissue sample.

The specific *BEST3* primer for qPCR analysis is listed in Table 6. The elongation factor gene (*EIF*) has been demonstrated to exhibit stable expression across various tissues and experimental conditions, and was therefore employed as an internal reference gene [54]. The amplification efficiencies of *Mn-BEST3* and *EIF* were confirmed to be approximately comparable, thereby allowing the application of the 2^−ΔΔCt^ method for the calculation of relative gene expression levels [55].

### 4.4. RNAi Analysis

To explore the potential regulatory function of *Mn-BEST3* in the growth performance of *M. nipponense*, an RNAi experiment was conducted. A total of 240 prawns were randomly divided into the control group (injected with *dsGFP*) and the experimental group (injected with *dsBEST3*). Each group comprised 60 males and 60 females. The *dsGFP* treatment served as a negative control to monitor non-specific effects [56]. The initial mean body weights were as follows: in the *dsGFP*-injected control group, males weighed 0.781 ± 0.11 g and females weighed 0.424 ± 0.05 g; in the *dsBEST3*-injected group, males weighed 0.780 ± 0.10 g and females weighed 0.417 ± 0.03 g.

Using the Snap Dragon tool (https://www.flyrnai.org/cgi-bin/RNAi_find_primers.pl, accessed on 7 June 2024), we designed RNAi primers that were specific to *BEST3* and flanked by T7 promoter sequences (listed in Table 6). For both the *Mn-BEST3* gene and the *GFP* control, double-stranded RNA (*dsRNA*) was produced in vitro with the Transcript Aid™ T7 High Yield Transcription Kit (Fermentas, Inc., Rockville, MD, USA), strictly adhering to the manufacturer’s instructions. Following a previously reported protocol [38], each prawn in the experimental cohort was microinjected with *dsBEST3* through cavum pericardial, whereas those in the control cohort were injected with *dsGFP*. The *dsRNA* solutions were adjusted to 4 µg/µL in an isotonic vehicle, and a dose of 4 µg per gram of body weight was administered to each animal. This dosing regimen meant that the injection volume in microliters for any given prawn was equal to its body weight expressed in grams. Both the experimental cohort and the control cohort received injections of *dsBEST3* and *dsGFP* once every 6 days, for a total of 3 injections. To monitor the effectiveness of RNAi, we measured *Mn-BEST3* transcript levels in muscle tissue via qPCR at four time points after injection: days 1, 6, 12, and 18 (with at least five individuals per time point). The procedures for RNA extraction, cDNA synthesis, and qPCR analysis were as described in detail in Section 4.2 and Section 4.3. On the same sampling days, body weight measurements were also taken for each prawn.

### 4.5. Identification of Growth-Related SNPs Within BEST3

Total RNA was first extracted from the muscle tissue of each experimental individual, from which cDNA templates were subsequently generated by reverse transcription, following the protocol detailed in Section 4.2. Using the resulting cDNA as templates, three pairs of primers (Table 3) were employed to amplify the target regions via PCR. The PCR amplification and sequencing procedures were performed identically to those described in Section 4.2. The obtained sequences were then assembled and aligned with MEGA version 11.0 [52], allowing for the identification of SNP loci within the *BEST3* gene according to the criteria of previous reports [18]. Observed heterozygosity (*Ho*), and expected heterozygosity (*He*), were calculated using Popgene32 software (version 1.32) [57]. Polymorphic information content (*PIC*) was calculated using *PIC_CALC* [58]. SPSS Statistics 23.0 was used to examine the *p*-value of each SNP locus, as well as the relationship between each SNP locus and two growth traits (body weight and total length). A one-way ANOVA was carried out, followed by post hoc comparisons using the least significant difference (LSD) and Duncan’s multiple range test. Before performing the ANOVA, the normality of the data was assessed using the Shapiro–Wilk test, and the homogeneity of variances was checked with Levene’s test. Body weight and total length were entered as dependent variables in the statistical models. A probability value of less than 0.05 was considered statistically significant.

### 4.6. Statistical Analysis

All statistical analyses in this study were performed with SPSS Statistics 23.0 [18]. To determine significant differences between groups (different mature tissues, *dsBEST3* silencing efficiency validation, and comparisons of mass percentage increase between *dsBEST3*- and *dsGFP*-injected prawns), a one-way analysis of variance (ANOVA) was applied, followed by multiple comparisons using both LSD and Duncan’s post hoc tests. Quantitative measurements are presented as mean values with their corresponding standard deviations (±SD). A probability value below 0.05 was considered statistically significant.

## 5. Conclusions

Collectively, based on the results of qPCR analysis in various mature tissues and RNAi analysis, BEST3 was identified as a positive regulator of growth performance in both sexes of *M. nipponense*. Notably, a SNP locus (S31_23192836) within the coding region of *BEST3* was also identified to be associated with growth performance. Specifically, in both male and female prawns, individuals carrying the T allele had significantly higher average body weight and total length compared to those with other genotypes. Taken together, these findings establish the regulatory role of *BEST3* in growth performance and underscore the utility of its growth-associated SNP for marker-assisted selection in *M. nipponense* breeding. In the present study, the growth-related SNP locus was validated only within a single full-sib family, which limits its general applicability. In future studies, the association between this SNP locus and growth will be examined across multiple populations of this species.

## Figures and Tables

**Table 1:** The positions of exons of *Mn-BEST3*.

Exon	Start	Stop	Length
1	23,166,703	23,166,707	5
2	23,170,018	23,170,152	135
3	23,175,994	23,176,160	167
4	23,178,894	23,179,051	158
5	23,192,714	23,192,866	153
6	23,193,631	23,193,711	81
7	23,194,232	23,194,383	152
8	23,199,015	23,199,145	131
9	23,206,832	23,206,991	160
10	23,210,194	23,210,245	52

**Table 2:** Daily weight and length gain of *M. nipponense* after knockdown of *BEST3*.

Gender	Group	Weight Gain Rate (‰)
Female	*dsBEST3*	3.179 ± 0.919 *
	Control	4.293 ± 1.831
Male	*dsBEST*	5.384 ± 0.274 *
	Control	6.993 ± 0.563

* indicated the significant difference between the *dsBEST3* and control group (*p* < 0.05).

**Table 3:** Identification of SNPs within the coding region of *Mn-BEST3*.

SNP	Genotype1	Genotype2	Genotype3	*Ho*	*He*	*PIC*	*p*-Value	Variation Type
S31_23176129	C:26	T:30	Y:36	0.379	0.393	0.316	0.262	Synonymous
S31_23192836	G:17	T:47	K:27	0.311	0.292	0.247	0.022	Synonymous
S31_23206944	A:25	C:31	M:36	0.396	0.358	0.294	0.114	Synonymous

**Table 4:** Identification of growth-associated SNPs within the coding region of *Mn-BEST3*.

SNP ID	Gender	Genotype (Number)	Weight (g)	Full Length (mm)
S31_23192836	Female	TT:22	1.117 ± 0.504 b	46.237 ± 6.100 b
		TG:14	0.897 ± 0.429 ab	43.692 ± 6.350 ab
		GG:10	0.500 ± 0.190 a	38.057 ± 5.155 a
	Male	TT:25	2.500 ± 0.682 b	59.532 ± 5.635 b
		TG:13	1.699 ± 0.910 ab	52.919 ± 8.475 ab
		GG:7	1.057 ± 0.142 a	47.097 ± 1.539 a

Letters indicated the significant difference between different genotypes (*p* < 0.05).

**Table 5:** Specimens used in this study.

Sampling Data	Animals	Tissue	Purpose	Body Weight
2023.07.04–07.07	10 specimens	Muscle	ORF verification	2.93–3.82 g
2023.07.04–07.07	18 male specimens and 18 female specimens	Eyestalk, Brain, Heart, Hepatopancreas, Gill, Muscle, Ovary, Testis	qPCR analysis	2.68–3.85 g for male prawns; 1.74–2.25 g for female prawns
2024.06.15–07.06	240 specimens (120 males and 120 females)	Muscle	RNAi analysis	0.76–0.82 g for male prawns; 0.41–0.44 g for female prawns
2024.09.12	100 speimens (50 males and 50 females) from a full-sib family	Muscle	SNP identification	0.86–3.89 g for male prawns; 0.36–1.58 g for female prawns

**Table 6:** Primers used in the present study.

Primer	Sequence	Product Length	Purpose
F1	CGGTGCTCTACTGTGATGCG	481 bp	Primers for PCR verification and SNP identification
R1	GCCATGAAAAAGGCGTAGGT		
F2	CACTCCTGCGGGACATCAAG	538 bp	
R2	TTACTTCTGGGGTTGAGGCG		
F3	TGGTAATCCCAGAAGACAAACAGA	303 bp	
R3	TCCTAATGCCCTTACTACCTGC		
RT-F1	GATAGCCCTACACGTCACGG	125 bp	Primer for qPCR
RT-F2	AACGCTTTTTGACAGCGGAC		
EIF-F1	CATGGATGTACCTGTGGTGAAAC	179 bp	Primer for reference gene
EIF-R1	CTGTCAGCAGAAGGTCCTCATTA		
RNAi-F1	TAATACGACTCACTATAGGGGGCCGGTTTTATCACTTCAA	443 bp	Primer for RNAi
RNAi-R1	TAATACGACTCACTATAGGGGGGAGGTGTCCGAATGAGTA		

## Data Availability

The original contributions presented in this study are included in the article. Further inquiries can be directed to the corresponding authors.
